# Assigning Backbone NMR Resonances for Full Length Tau Isoforms: Efficient Compromise between Manual Assignments and Reduced Dimensionality

**DOI:** 10.1371/journal.pone.0034679

**Published:** 2012-04-18

**Authors:** Nicholas W. Harbison, Shibani Bhattacharya, David Eliezer

**Affiliations:** 1 Tri-Institutional Program in Chemical Biology, Department of Biochemistry, Weill Cornell Medical College, New York, New York, United States of America; 2 New York Structural Biology Center, New York, New York, United States of America; Spanish National Cancer Center, Spain

## Abstract

Tau protein is the longest disordered protein for which nearly complete backbone NMR resonance assignments have been reported. Full-length tau protein was initially assigned using a laborious combination of bootstrapping assignments from shorter tau fragments and conventional triple resonance NMR experiments. Subsequently it was reported that assignments of comparable quality could be obtained in a fully automated fashion from data obtained using reduced dimensionality NMR (RDNMR) experiments employing a large number of indirect dimensions. Although the latter strategy offers many advantages, it presents some difficulties if manual intervention, confirmation, or correction of the assignments is desirable, as may often be the case for long disordered and degenerate polypeptide sequences. Here we demonstrate that nearly complete backbone resonance assignments for full-length tau isoforms can be obtained without resorting either to bootstrapping from smaller fragments or to very high dimensionality experiments and automation. Instead, a set of RDNMR triple resonance experiments of modest dimensionality lend themselves readily to efficient and unambiguous manual assignments. An analysis of the backbone chemical shifts obtained in this fashion indicates several regions in full length tau with a notable propensity for helical or strand-like structure that are in good agreement with previous observations.

## Introduction

First isolated as microtubule-associated proteins [1], the tau protein family is comprised predominantly of six isoforms that are alternately spliced from a single gene [2], [3]. Localized chiefly to the axons in both the central and peripheral nervous systems tau functions primarily to facilitate and stabilize microtubule polymerization [4]–[6]. Aside from its primary role, tau is implicated in many different cellular processes including axon development [7], signal transduction [8] and mediation of microtubule-membrane interactions [9]. Perhaps one of the most important cellular functions of tau is to mediate signals for various kinases. There are numerous phosphorylation sites within tau that help regulate its many functions, including binding to microtubules [10]–[13].

While tau plays many roles in normal cellular function it is most studied for its role in neurodegeneration. Tau is the primary component in neurofibrillary tangles (NFT), a pathological hallmark of a group of diseases called tauopathies that includes Alzheimer's disease (AD), progressive supranuclear palsy and Pick's disease among others [14]. The core of NFT is comprised solely of tau in various aggregate forms including, paired helical filaments and straight filaments [15], [16]. Though the role of these filaments in the progression of the diseases remains unclear, genetic mutations in the tau gene were discovered in patients suffering from frontotemporal dementia and parkinsonism (FTDP) [17], [18], demonstrating that tau plays a more direct role in neurodegeneration than originally thought.

**Figure 1:**
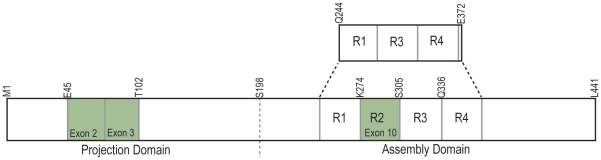
Diagram of the full-length tau protein, Tau441 showing the domain structure, alternately spliced exons (green) and the microtubule-associated repeats (R1–R4). Tau352 is the shortest full-length form of tau with no alternately spliced regions. TauK19 containing R1, R3 and R4 is shown above.

There are two general domains in the primary structure of tau. The N-terminal or projection domain protrudes from the microtubule surface while the C-terminal or assembly domain, which contains either three or four semi-conserved microtubule-binding motifs, remains associated with the microtubule surface [19]. The six distinct isoforms are formed from alternate splicing of exons 2 and 3 in the N-terminal domain, and exon 10 in the microtubule-associated region (Fig. 1).

In solution tau is an intrinsically disordered protein (IDP), lacking a well-defined tertiary structure. Since IDPs do not form compact well-ordered structures they cannot be typically crystallized in isolation. In contrast, nuclear magnetic resonance (NMR) spectroscopy has proven particularly useful for the study of IDPs in solution [20], [21]. Indeed numerous NMR studies have looked at the structure of the various isoforms of tau and their cellular interactions [11], [22], [23]. For example, we have previously studied the properties of a microtubule-binding repeat containing fragment of tau (Tau K19) both in solution and when interacting with lipid surfaces [24]. Subsequently, a number of NMR studies of tau fragments [25], [26] and more recently of full length tau isoforms [27]–[29] have been reported. Due to the unstructured nature of the protein NMR spectra of tau protein have sharp resonances but lack the high degree of spectral dispersion characteristic of well-ordered proteins. This causes resonance overlap which becomes even more pronounced when trying to study the full-length isoforms (Tau441). One way to overcome the resonance overlap in spectra of longer tau isoforms is to selectively label specific amino acids to obtain partial spectra that can be added together [30]. A second method involves the use of splicing information extracted from spectra collected using smaller fragments of the protein [31]. A third method is to use higher dimensionality experiments, which however can demand prohibitive NMR data acquisition times. A solution to this problem has appeared in the past few years in the form of reduced dimensionality NMR experiments (RDNMR), which permit for the acquisition of high-dimensionality, and therefore well-dispersed, NMR data using shorter acquisition times. Most recently, very high dimensionality (6D and 7D) RDNMR data were used in combination with automated analysis to produce backbone resonance assignments for full length tau [32]. Here we report the use of lower dimensionality RDNMR experiments combined with manual analysis to accomplish the same goal. Manual analysis is difficult with very high dimensionality data, yet is often desirable for intervention, verification, and correction of assignments.

We employed G-Matrix Fourier Transformation NMR (GFT-NMR), one of several recently developed reduced-dimensionality approaches to acquiring NMR data that uses phase-sensitive joint sampling of the indirect dimension to produce subspectra onto which a G-matrix is applied prior to fourier transformation [33], [34]. GFT has been applied to several experiments to allow for higher-dimensional data acquisition through the mathematical linear combinations of the subspectra [35]–[37]. We report the use of both established and new GFT acquisition sequences (unpublished) to obtain de novo resonance assignments for three tau constructs: tau K19, Tau352 (full length tau with all alternatively spliced regions absent), and Tau441 (the longest tau isoform), without the use of bootstrapping assignments obtained from small fragments [31] or of fully automated computational analyses [32]. The method presented here is a good compromise between these two previously demonstrated approaches.

## Methods

### Samples

Recombinant proteins were expressed in *Escherichia coli* cells transfected with plasmids for TauK19 (gift from Drs. Peter Lansbury and Kenneth Kosik), Tau352 or Tau441 (gifts from Dr. Guy Lippens), all under the control of a T7 promoter. The FTDP-linked point mutation K257T was introduced into the TauK19 isoform using a stratagene mutagenesis kit. The proteins were purified as follows: cells were lysed by sonication in a solution containing 3 M Urea, 1 mM EDTA, 1 mM DTT, 1 mM PMSF and 10 mM Tris at pH 8.0 followed by ultracentrifugation at 40,000 rpm in a Beckman ultracentrifuge using a Ti 50.2 rotor. The supernatant was further purified using a streptomycin sulfate precipitation before being dialyzed against 25 mM Tris, 20 mM NaCl, 1 mM EDTA and 1 mM DTT and applied to a cation-exchange column and eluted by a NaCl gradient. Fractions containing tau were pooled, dialyzed against 5% acetic acid and purified on either a C18 (TauK19) or C4 (Tau352, Tau441) reverse-phase HPLC column using an acetonitrile gradient with 1% trifluoroacetic acid. The purified protein was lyophilized and stored at −20°C. Purity was verified by SDS-PAGE.

NMR samples for TauK19 were prepared by resuspending lyophylized protein in PBS buffer (100 mM NaCl and 10 mM Na2HPO4 with 10% D20 at pH 7.4) and samples for larger isoforms in Tris buffer (REF) (25 mM Tris, 50 mM NaCl, 2.5 mM EDTA, 5 mM DTT, 5% D20 at pH 6.8).

### Data Collection

All spectra were collected on Bruker *AVANCE* 600 and 900 MHz spectrometers equipped with z-axis gradient TCI CryoProbes (New York Structural Biology Center). For backbone resonance assignments of each protein a series of (4,3) GFT experiments were acquired in conjunction with standard suite of triple resonance experiments [38]. Details of the experiments are provided in Table 1. All of the experiments were collected with 1024 points, a spectral width of 10 ppm and an offset of 4.7 ppm in the ^1^H dimension.

**Table 1:** Acquisition Parameters of GFT-NMR Experiments.

Experiment name	Linear combination of shifts (relative to carriers) measured in GFT	TauK19 † Dimension: complex points/sw (ppm)	Tau352 § Dimension: complex points/sw (ppm)	Tau441 § Dimension: complex points/sw (ppm)
‡(4,3)D H^N^NC^αβ^C^α^	Ω_0_(^13^C^α^_i/i−1_) ± Ω_1_(^13^C^α^_i/i−1_) Ω_0_ (^13^C^α^_i/i−1_) ± Ω_1_(^13^C^β^_i/i−1_)	**^15^N**:25/23 **^13^C**:55/80	^1**5**^ **N**:50/23 **^13^C**:64/80	^1**5**^ **N**:50/23 **^13^C**:64/80
‡(4,3)D C^αβ^C^α^(CO)NH^N^	Ω_0_(^13^C^α^_i−1_) ± Ω_1_(^13^C^α^_i−1_) Ω_0_ (^13^C^α^_i−1_) ± Ω_1_(^13^C^β^_i−1_)	**^15^N**:20/23 **^13^C**:50/80	**^15^N**:50/23 **^13^C**:64/80	**^15^N**:50/23 **^13^C**:64/80
(4,3)D H^N^NC^α^CO	Ω_0_(^13^C^′^_i_) ± Ω_1_(^13^C^α^_i_) Ω_0_(^13^C^′^_i−1_) ± Ω_1_(^13^C^α^_i−1_)	**^15^N**:25/23 **^13^C**:75/65	**^15^N**:36/23 **^13^C**:75/60	Did not collect
‡(4,3)D H^N^NCOC^α^	Ω_0_(^13^C^′^_i−1_) ± Ω_1_(^13^C^α^_i−1_) Ω_0_(^13^C^α^_i−1_) ± Ω_1_(^13^C^′^_i−1_)*	**^15^N**:25/23 **^13^C** *:75/65	**^15^N**:36/23 **^13^C**:75/60	Did not collect
(4,3)D H^N^N(C^α^)NH^N^	Ω_0_(^15^N_i+1_) ± Ω_1_(^1^H_i+1_) Ω_0_(^15^N_i−1_) ± Ω_1_(^1^H_i−1_)	Did not collect	**^15^N (t2)**:32/23 **^15^N (t3)**:55/40	**^15^N (t2)**:32/23 **^15^N (t3)**:55/40
(4,3)D H^N^N(COC^α^)NH^N^	Ω_0_(^15^N_i+1_) ± Ω_1_(^1^H_i+1_)	Did not collect	**^15^N (t2)**:32/23 **^15^N (t3)**:55/40	Did not collect

† Data collected at 600 MHz.

§ Data collected at 900 MHz.

‡ Experimental details published in reference [35].

* For this experiment Ω(C^α^) was detected in quadrature.

### Data Analysis

Data was processed with NMRPipe and analyzed using NMRViewJ. To prepare the secondary chemical shift plots, C^α^ chemical shift values were extracted from both the additive and subtractive (4,3)D H^N^N **C** ^αβ^ **C** ^α^ experiments. These values were then averaged to give the correct chemical shift for each residue. The expected random coil shift for each residue was then subtracted from the experimental chemical shift to obtain the secondary shift.

## Results

**Figure 2:**
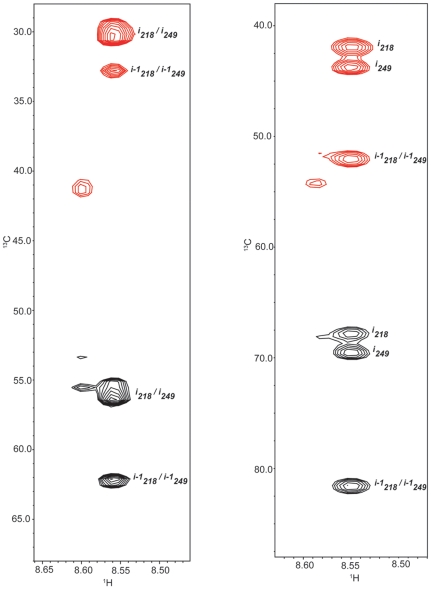
^13^C/^1^H strip plots from 3D (left) and (4,3)D (right; additive experiment) HNCACB experiments, at ^15^N chemical shifts of 125.69 ppm and 125.67 ppm, respectively, for Tau K19 K257T. Shown are the spin systems for residues Gln307 and Glu338 (corresponding to tau352 residues 218 and 249) which can be better and more accurately resolved and identified using the GFT linear combinations. Positive peaks are shown in black and negative peaks are in red.

**Figure 3:**
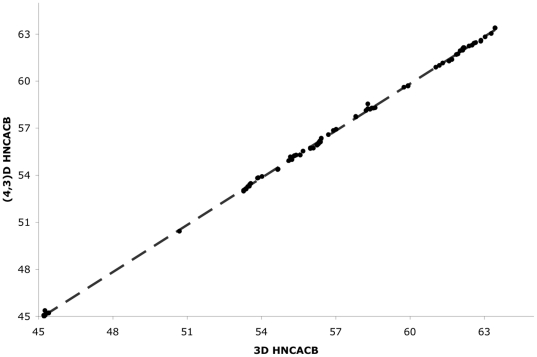
Correlation plot of the C^α^ chemical shift values from the (4,3)D HNCACB experiment versus those from the standard 3D HNCACB for tauK19 K257T. The lease squares fit has a slope of 1.001 and an R^2^ value of 0.999.

In order to test the efficacy of applying GFT to the tau system, (4,3)D H^N^NC^αβ^C^α^ and (4,3)D C^αβ^C^α^(CO)NH^N^ spectra were acquired, at 600 MHz, for tau K19 with the K257T mutation enabling assignments for 98% of ^1^HN, ^15^N and C^αβ^ atoms for this construct. One of the major advantages in using the GFT-based experiments is that many peaks that were difficult to resolve in the standard 3D HNCACB are now better differentiated (Fig. 2) allowing for an increased level of both precision and confidence in the assignments [33]. This results from the fact that in general a linear combination of the chemical shifts of several nuclei is less likely to be degenerate between residues as illustrated in Figure 2. In non-GFT multidimensional experiments typically the resolution of overlapped peaks in the indirect dimensions is limited by the digital resolution, which becomes a significant issue for data acquired at higher fields. The overall agreement between the 3D and (4,3)D data is shown by a linear fit on a correlation plot of the chemical shifts obtained from the GFT versus standard HNCACB experiments. The plot shows good agreement between the GFT values with only minor deviations seen in the aspartic acid region (Fig. 3).

**Figure 4:**
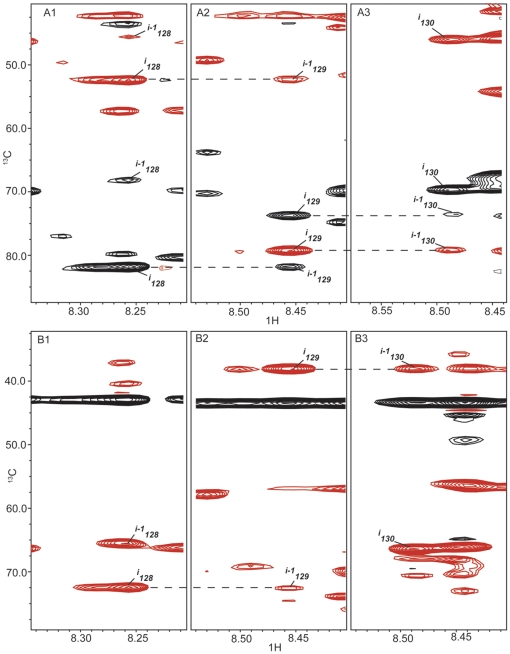
^13^C/^1^H strip plots showing inter-residue connectivities for residues Val128, Ser129, and Lys130 from the (4,3)D HNCACB additive (A) and subtractive (B) data for Tau352 at, at ^15^N chemical shifts of (1) 122.31 ppm (2) 120.14 ppm and (3) 124.10 ppm. Assignments were made by walking through the data from *i* to *i−1* spin systems. Positive peaks are shown in black and negative peaks are in red. Dashed lines show peak connectivities.

**Figure 5:**
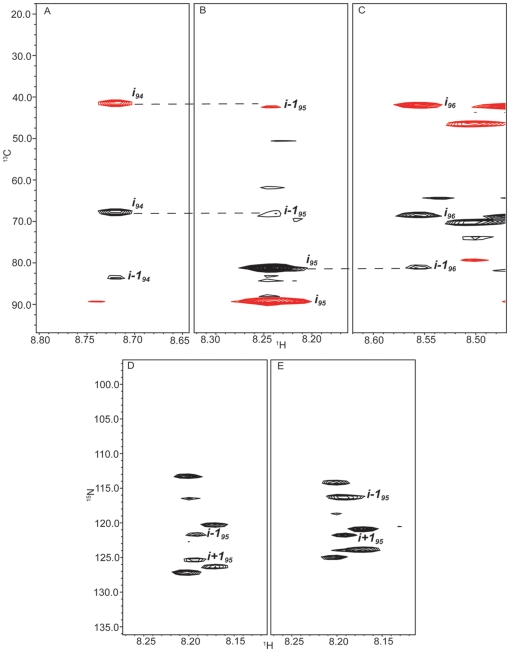
^13^C/^1^H strip plots at ^15^N chemical shifts of 119.06 ppm (A), 116.36 ppm (B), and 123.59 ppm (C) extracted from the (4,3)D HNCACB experiment for Tau441 (only the additive strips are shown) corresponding to residues His94, Thr95, and Glu96. Dashed lines indicate the inter-residue connectivities. Assignments were verified using the additive (D) and subtractive (E) linear combinations of the (4,3)D HNNCANNH data as shown by ^15^N/^1^H strip plots below at the ^15^N chemical shift of Thr95 at 116.58.

**Figure 6:**
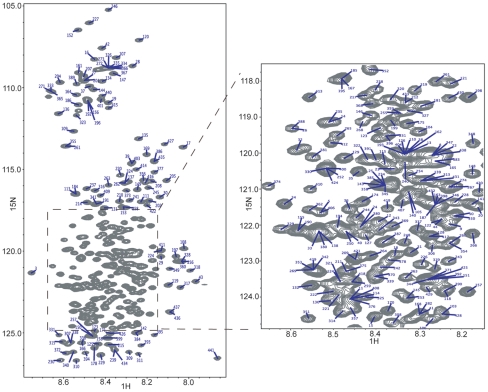
^15^N/^1^H HSQC spectrum for free state tau352 with peak assignments given by residue numbers for the longest tau isoforms (tau441). The highly overlapped region (box) is enlarged.

Having confirmed that the K19 data from the GFT based experiments proved reliable, we collected an expanded set of GFT experiments at 900 MHz for the shortest and longest physiological tau isoforms, Tau352 and Tau441. Full assignments for Tau352 were initially made by “walking” through the (4,3)D H^N^NC^αβ^C^α^ spectra (Fig. 4). Due to the high level of sequence redundancy coupled to the random coil structure in tau, just utilizing this standard practice did not provide an unambiguous set of assignments. We then refined the assignments further by using a linear combinations of amide nitrogen and proton shifts obtained from the intra- and inter-residue connectivities in the (4,3)D H^N^N(C^α^)NH^N^ and (4,3)D H^N^N(COC^α^)NH^N^ experiments respectively (Table 1). The ability to correlate the ^15^N chemical shift of the *i^th^* residue with those of the *i−1* and *i+1* residues in the HNCANNH type experiments [39] provides a crucial link in the assignment strategy of unfolded and/or large proteins [40]. After splitting the GFT data two distinct resonances, one for *i−1* and one for *i+1*, are present for each *i^th^* residue in the two subspectra. Once the *i+1* peak has been identified from the (4,3)D H^N^N(COC^α^)NH^N^ experiment the ^15^N chemical shift for residue *i−1* in the f3 dimension (in ppm) is extracted simply by averaging the corresponding *i−1* 15N shifts from the sub-spectra. For example, in Figure 5 the position in the f3 dimension of the peaks corresponding to the *i−1* residue (His94) are at 121.71 ppm and 116.34 ppm for the additive (panel D) and subtractive (panel E) combinations respectively. Averaging these values yields a ^15^N chemical shift of 119.03 which corresponds exactly to that of His94 (119.06 PPM) in the (4,3)D H^N^NC^αβ^C^α^ experiment (panels A–C). The same holds true for *i+1* peak with ^15^N shift of 123.56 ppm calculated from GFT data matching the C^αβ^ correlations at 123.59 ppm in the nitrogen dimension of the (4,3)D H^N^NC^αβ^C^α^ experiment. By applying the N15 chemical shifts to build and confirm overlapping fragments comprised of *i−1, i*, and *i+1* residues we were successful in eliminating much of the uncertainty in assignments that arose, primarily, from sequence overlap and allowing for 97% completion of backbone assignments of Tau352 (Fig. 6).

**Figure 7:**
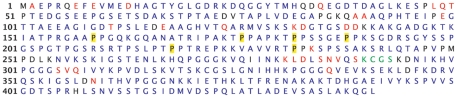
Primary sequence of Tau441 showing residues with complete C^α^, HN, N assignments in blue and residues with HN and C^α^ or HN and N assignments in red. Residues in green can be logically assigned based on sequence overlap while the prolines highlighted in yellow cannot be assigned using these experiments.

With confidence in this assignment strategy we moved on to making assignments for Tau441. The presence of all three alternately spliced exons increases the spectral overlap considerably though the quality of the data remains high. Following the same assignment strategy as with Tau352, using data from (4,3)D H^N^NC^αβ^C^α^ and (4,3)D H^N^N(C^α^)NH^N^ experiments to determine the spin system connectivities, we were able to obtain 85% complete assignment of the backbone resonances ^1^HN, ^15^N and C^α^ for each residue (not counting the leading proline in pro-pro couplets, which can be assigned manually using other experiments, as noted in [32]), though that figure increases to 92% when including partial assignments of ^1^HN and either ^15^N or C^α^ chemical shifts (Fig. 7). Some partial assignments result from sequence repetition (i.e Ala89-Ala91) though most are caused by spectral overlap in one of the spectra, especially when dealing with charged residues (Asp, Glu, Lys).

**Figure 8:**
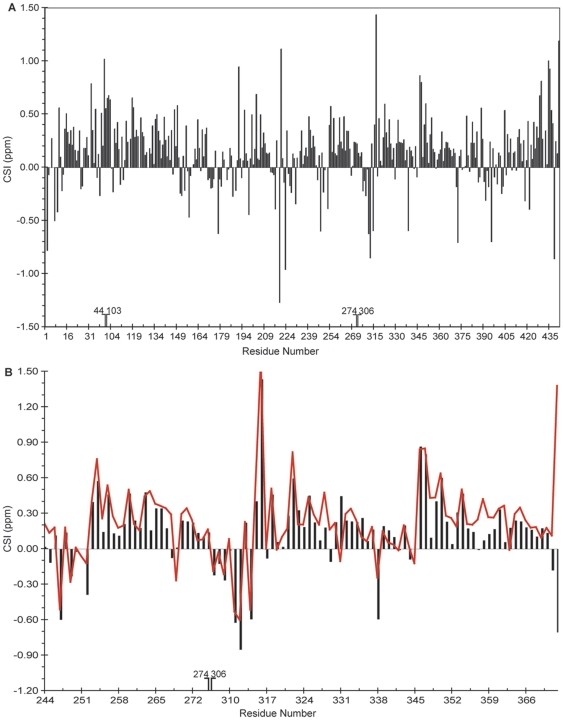
CSI plots for Tau352 (A) full sequence and (B) microtubule binding region (bars) overlaid with data for TauK19 (red line). Positive deviations are indicative of α-helical conformations while negative deviations correspond to β-sheet structure.

Though tau is unstructured in solution, residual secondary structural propensities can be determined by plotting the secondary chemical shifts or the difference between the experimental chemical shift and the expected random coil value. In particular, C^α^ secondary chemical shifts are very sensitive to the φ and ψ dihedral angles and are good reporters of the protein secondary structure [41], [42]. Positive shifts are indicative of α-helical propensity while negative shifts represent β-strand or extended elements. Figure 8B shows the secondary shifts derived from our current GFT data for tau K19, plotted together with secondary shifts from previously published studies of the K19 construct [24]. The two sets of secondary shifts are in good agreement, and both show the originally noted three regions of positive secondary shifts indicating three regions of helical propensity, confirming the accuracy of the GFT-derived shifts. Figure 8A shows the secondary shifts for Tau352. As we can see, in addition to the regions of helical propensity previously noted in the microtubule-binding repeats of K19, several other regions of tau 352 exhibit significant groupings of positive secondary shifts, indicating a preference for helical formations. In addition, as might be expected, there are some regions of negative shifts around residues 151–158, 170–189, and 214–230 which are all found in the proline-rich region of the protein, as well as at residues 306–311, which correspond, as previously noted, to the VQIVYK PHF6 motif originally identified by the Mandelkows [43].

## Discussion

NMR has long been a valuable tool in the study of protein structure, but is particularly valuable in the study of non-native protein states and especially of intrinsically disordered proteins – i.e. those that do not form well-defined native structure when isolated in solution. A well-known representative of this class of proteins is the microtubule-associated protein tau, which is of particular interest for its role in several neurodegenerative diseases, including Alzheimer's disease and Frontemporal Dementia. Despite being a relatively large protein, containing 441 residues in its longest form, tau contains no well-structured domains, and the entire polypeptide is highly dynamic and disordered in solution. Tau has been the subject of NMR studies for several years, mostly applied to smaller fragments of the protein, but only recently has technology advanced sufficiently to allow the study of full-length tau constructs in more detail. Previous studies of the full-length protein reported partial backbone NMR resonance assignments of full-length constructs of tau obtained using selective labeling of specific amino acids [30] or full assignments obtained by bootstrapping from or splicing assignments of smaller constructs [31]. More recently, an automated assignment method using very high dimensionality (6D and 7D) experiments acquired using RDNMR methods such as automatic projection reconstruction spectroscopy (APSY) was reported allowing nearly complete backbone assignments to be obtained that compared well with the previously reported results [32].

Here we report the use of RDNMR (specifically of G-matrix fourier transform techniques) with a relatively modest dimensionality (4D) to obtain *de novo* backbone assignments for both the shortest and longest physiological tau isoforms: Tau352 and Tau441. Using two experiments acquired over only a few days we are able to make reliable protein assignments for 97% of Tau352 and 92% of Tau441 in a matter of weeks. The results are comparable (93% assignment reported) to the computer-aided APSY method, and this approach is considerably more efficient than the originally reported fragment bootstrapping method, which took several months to years to obtain and required many experiments on many constructs. Our approach facilitates manual intervention where necessary to check connectivities, resolve ambiguities, and confirm and correct assignments. Owing to lower S/N of larger proteins and spectral artifacts, the general reliability of automatically generated assignments based on higher dimension data has not been established with absolute certainty, although excellent results obtained for the case of tau [32].

Comparison of the GFT-NMR generated chemical shifts and secondary shifts with data previously published from standard experiments (Fig. 8B) shows an excellent level of agreement confirming the accuracy of shifts obtained using the GFT method. Indeed, GFT methods may be expected to provide more accurate shifts since the linear combinations increase the dispersion of the data and thereby decrease overlap. Our C^α^ secondary shifts calculated using the GFT-based assignments suggest that several regions of tau beyond the formerly identified microtubule-binding repeat regions exhibit a preference for helical structure. These include the C-terminal ∼20 residues of the protein, which was also observed to populate helical conformations in a study of a 187- residue C-terminal fragment of the protein [25], short stretches around residues 200 and 240, and about 70 residues at the N-terminal end of the molecule starting at residue ∼20. Helicity at position 240 has been observed in studies of a fragment containing this region (unpublished data) and is stabilized by CDK2/CycA3 phosphorylation [44]. Helicity in the N-terminal region is evident in the analysis of the bootstrapped assignments of the full length protein [29]. Interestingly, the I1 and I2 alternatively spliced inserts interrupt the relatively contiguous helical propensity observed for tau352, suggesting that splicing can influence the structural properties of tau. Evidence in our data for extended or β-sheet structure is strongest at the previously identified PHF6 motif, as well as in the polyproline region, between the microtubule binding repeats and the helix preferring C-terminal region, and at the very N-terminus of the molecule, again consistent with observations made using the bootstrapped assignments.

Obtaining residue-specific NMR backbone resonance assignments is a crucial step in the detailed structural characterization of disordered proteins. The chemical shifts obtained from assignments of full-length tau isoforms report directly on the secondary structure propensities of the protein as described above and in previous reports [29]. Furthermore, the availability of such assignments has resulted in important new information regarding the interaction of the protein with microtubules [45], the effects of phosphorylation [44], [46], and key features in tau aggregation [47]. Our results demonstrate a rapid and accessible method, avoiding either laborious studies of multiple sub-fragments or highly automated methods employing less accessible very high dimensionality data, easily executed on any modern spectrometer and requiring minimal computing power, for obtaining such assignments for large disordered proteins.
